# Proteomic Strategies to Evaluate the Impact of Farming Conditions on Food Quality and Safety in Aquaculture Products

**DOI:** 10.3390/foods9081050

**Published:** 2020-08-04

**Authors:** Mónica Carrera, Carmen Piñeiro, Iciar Martinez

**Affiliations:** 1 Food Technology Department, Institute of Marine Research (IIM), Spanish National Research Council (CSIC), 36208 Vigo, Pontevedra, Spain; 2 Scientific Instrumentation and Quality Service (SICIM), Institute of Marine Research (IIM), Spanish National Research Council (CSIC), 36208 Vigo, Pontevedra, Spain; cpineiro@iim.csic.es; 3 Research Centre for Experimental Marine Biology and Biotechnology—Plentzia Marine Station (PiE), University of the Basque Country UPV/EHU, 48620 Plentzia, Spain; iciar.martinez@ehu.eus; 4 IKERBASQUE Basque Foundation for Science, 48013 Bilbao, Spain

**Keywords:** proteomics, discovery, target, aquaculture, mass spectrometry, dietary management, fish welfare, stress, food safety, antibiotic resistance

## Abstract

This review presents the primary applications of various proteomic strategies to evaluate the impact of farming conditions on food quality and safety in aquaculture products. Aquaculture is a quickly growing sector that represents 47% of total fish production. Food quality, dietary management, fish welfare, the stress response, food safety, and antibiotic resistance, which are covered by this review, are among the primary topics in which proteomic techniques and strategies are being successfully applied. The review concludes by outlining future directions and potential perspectives.

## 1. Introduction to Proteomics in Aquaculture

Aquaculture is the breeding of aquatic organisms under controlled conditions, involving both marine and freshwater fish along with algae, crustaceans, and mollusks. According to the Food and Agriculture Organization of the United Nations (FAO), this food sector represents a significant source of nutrients for the human diet and produces approximately 97.2 million tons of fish annually, which represents 47% of the global fish production [1]. With nine billion people expected to be living on the planet by 2050, maintaining the current level of fish consumption (9.0–20.2 kg annually per capita) is a challenging task [1]. Aquaculture, the most rapidly growing food-producing sector in the world, offers an excellent source of high value food and is expected to significantly contribute to meeting this demand for fish products.

The globalization of aquaculture markets presents important nutritional and economic benefits but also poses potential risks for food safety, such as the fraudulent substitution of fish species and the presence of food microorganisms, viruses, parasites, and vectors of their corresponding foodborne diseases [2]. Moreover, fish and seafood are easily spoiled, resulting in a fast loss of food quality due to the presence of fish microbiota, the elevated amount of unsaturated fatty acids and the abundance of proteases. Improving aquaculture practices to offer products of optimal quality and to reduce chronic stress throughout improved farming conditions to maintain fish welfare are two major questions in aquaculture research. Consumer awareness and a rising demand for aquaculture products have motivated scientists to develop procedures to enhance productivity and to improve the quality and safety of these foodstuffs. In this context, proteomics has been established as a powerful methodology for the evaluation of quality and safety in aquaculture products [3,4,5].

Proteomics is the high-throughput analysis of the proteins of a specific biological sample [6]. Proteomics involves the identification, localization and quantification of proteins as well as the analysis of protein modifications and the elucidation of protein-protein networks [7]. Among proteomic analytical techniques, mass spectrometry (MS) is recognized as an indispensable instrument to precisely analyze a large number of proteins from complex samples in the majority of food proteomics studies [8,9]. Additionally, the computational analysis of MS data has improved the discriminatory power of proteomics techniques, making them effective methodologies for the global analysis of proteins and peptides [10]. Thus, the latest advances in proteomics and bioinformatics approaches have turned them into useful tools to develop promising strategies for food science investigations [11,12]. Within that framework, the present review summarizes some highly relevant applications of proteomics to evaluate the impact of farming conditions in fish wellbeing as well as the quality and safety of aquaculture products.

## 2. Workflow of Proteomics: Discovery and Targeted Proteomics

**Figure 1:**
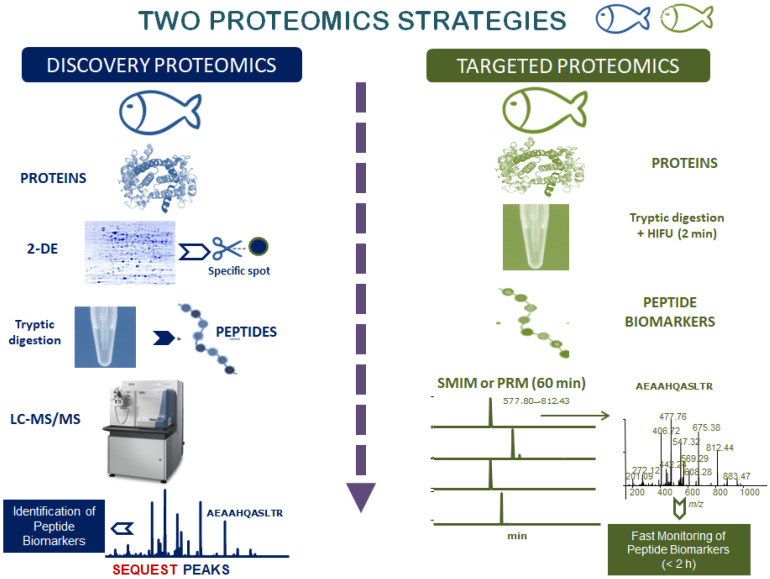
Workflow of proteomics: discovery and targeted proteomics.

Proteomics has the potential to provide information useful to improve the production, welfare, health, nutritional value and wholesomeness of farmed fish. Figure 1 shows the classical proteomics approaches, i.e., discovery and targeted proteomics, with their corresponding workflows.

Discovery proteomics aims at identifying biological markers in a given proteome, frequently employing a bottom-up approach, in which the proteins of the sample are separated, proteolyzed with enzymes such as trypsin or Glu-C and the peptides obtained are subsequently analyzed by tandem mass spectrometry (MS/MS). Two-dimensional gel electrophoresis (2-DE) has traditionally been the technique selected for the separation of proteins samples [13]. This gel-based procedure is the most suitable approach for species whose protein sequences are not yet known, which includes many fish. In these cases, identification is performed by comparison of the MS/MS spectra of the peptides obtained with orthologous protein sequences from related species or by de novo MS/MS sequencing [14]. The 2-DE gels themselves can be analyzed by programs such as Progenesis and PDQuest.

In gel-free approaches, also known as shotgun proteomics, the proteins are directly digested in the extract with a selected enzyme, and the obtained mixture of peptides is subsequently analyzed by liquid chromatography (LC) coupled to tandem mass spectrometry (LC-MS/MS) [15,16]. It is possible to perform multidimensional LC separations, combining, for example, strong anion/cation exchange chromatography (SA/CX) and reverse phase (RP) chromatography [17]. Database searching programs, like SEQUEST, X! Tandem, or Mascot [18,19], allow the tentative identification of presumed peptide sequences based on the obtained fragmentation spectra, and additional software programs, such as Percolator are used to validate the identification [20]. When the protein is not present in the database, then the peptides must be sequenced de novo [21], either manually or using programs such as PEAKS and DeNovoX [22,23]. This approach has been successfully used in the de novo sequencing of some fish allergens, such as parvalbumins and shrimp arginine kinases [14,24,25]. When protein quantification is deemed necessary, the methods of choice include metabolic stable isotope labeling (such as stable isotope labeling by/with amino acids in cell culture, SILAC) [26]; isotope tagging by chemical reaction, such as isobaric tags for relative and absolute quantitation (iTRAQ), tandem mass tag (TMT) and difference gel electrophoresis (DIGE) [27,28,29]; stable isotope incorporation via enzyme reaction (i.e., ^18^O) [30]; and label-free quantification (i.e., measuring the intensity of the peptides at the MS level) [31]. After matching the obtained peptides and proteins by alignment software programs like BLAST (https://blast.ncbi.nlm.nih.gov/), it is possible to select relevant peptide biomarkers to be used in the subsequent phase namely, targeted proteomics.

Targeted proteomics refers to the monitoring of the relevant peptide biomarkers and it has become a recognized methodology to detect selected proteins with significant accuracy, reproducibility, and sensitivity [32]. In targeted proteomics, the MS analyzer is focused on detecting only the peptide/s chosen by selected/multiple-reaction monitoring (SRM/MRM) [33]. Monitoring appropriate transitions (evens of precursor and fragment ions *m/z*), represents a common analysis for detecting and identifying peptide biomarkers. These techniques are selective, sensitive, highly reproducible, with a high dynamic range and an excellent signal-to-noise (S/N) ratio [34]. SRM/MRM modes are usually performed on triple quadrupole (QQQ) instruments. This method possesses a highly sensitive scanning procedure but its optimization for a final SRM/MRM analysis is very time-consuming and, most importantly, this scanning mode does not produce entire MS/MS spectra. Since the spectrum of a peptide is critical to verify its sequence, new procedures are being used to obtain entire structural information; for instance, SRM-triggered MS/MS using hybrid quadrupole-ion trap (Q-IT) mass spectrometers, selected MS/MS ion monitoring (SMIM), parallel reaction monitoring (PRM) in IT or high-resolution Q-Orbitrap instruments are alternative targeted modes that enable the monitoring of precise peptides [35,36,37]. The development of targeted data independent analysis (DIA), conducted on a sequential windowed acquisition of all theoretical fragment ion spectra (SWATH-MS) [38], can identify and quantify thousands of proteins without the prerequisite of specifying a group of proteins prior to analysis. Stable-isotope dilution, ^13^C- or ^15^N-labeled absolute quantification peptide standards (AQUA) or concatemer of standard peptides (QCAT) can also be introduced to the sample as internal standards for absolute quantification of the proteins [39]. Programs such as SRMCollider and Skyline are accessible for the analysis of different targeted proteomic modes [40,41]. The following sections will show the application of the scanning mode for the follow up of peptide biomarkers identified in the discovery phase to assess the impact of farming conditions on food quality and safety of farmed fish.

## 3. Application of Proteomics to Evaluate the Farming Conditions on Food Quality and Safety in Aquaculture Products

**Figure 2:**
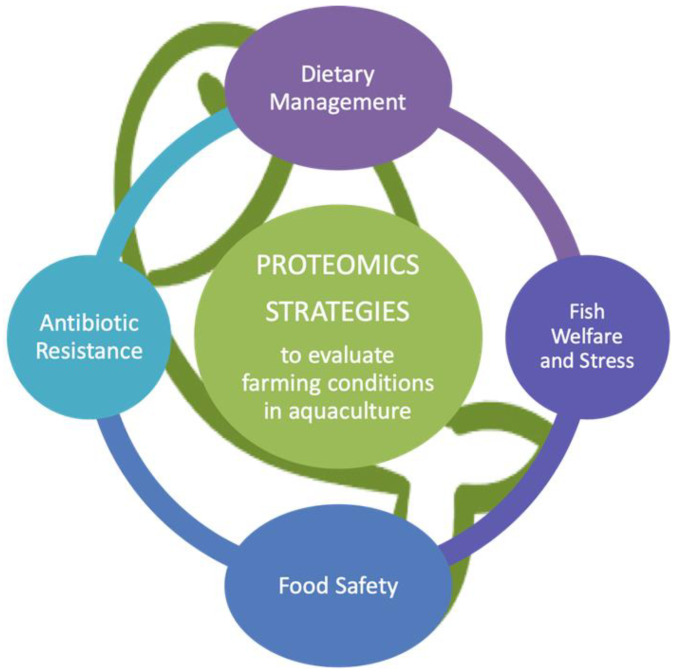
Summary of the main applications of proteomics techniques to evaluate the farming conditions in aquaculture reviewed in this publication.

Fish farming environments and conditions are very different from the conditions in which fish live in nature. Farmed fish, for instance, do not need to actively swim to catch their prey or escape predators; therefore, they exercise less, which impacts on their muscle growth and phenotype. The heavily processed feed consumed by farmed fish differs considerably from their natural diet, which affects muscle metabolism and biochemical composition. In addition, the farming conditions in aquaculture may not be optimized regarding stocking densities, incidence of parasites and diseases, and establishment of hierarchies due to competition for space or feed, all of which have consequences for the wellbeing and development of abnormal behavior. All these variables exert a strong influence on the yield, quality and wholesomeness of farmed seafood. Moreover, development of analytical methods to ensure that fish was farmed minimizing stressful factors is also of high relevance to satisfy consumer demands and labeling on the welfare of fish to be used for food. To investigate all these topics, powerful proteomic methodologies (discovery and targeted proteomics) may have a considerable impact on the understanding of current aquaculture practices in several major areas: (i) dietary management, (ii) fish welfare and response to stress, (iii) food safety, and (iv) antibiotic resistance (Figure 2).

### 3.1. Dietary Management in Aquaculture

Dietary management in aquaculture attempts to improve growth performance, health and immune status in living aquaculture organisms. Numerous works have shown that the composition of the feed influences the composition of fish fillet. Initial works using 2-DE revealed differences between the proteomes of skeletal muscle samples from wild and farmed fish. Carpene et al. [42], found differences by 2-DE in the level of abundance of the fast skeletal myosin light chain type 3 which seemed to be more abundant in wild than in farmed fish and, surprisingly, was also present in the red muscle of farmed, but not of wild, fish [42]. The 2-DE protein pattern of skeletal muscle of cod excised within 5 h of death revealed the presence of spots in the ranges of molecular weight between 35 and 45 kDa and between 50 and 100 kDa in the muscles of the farmed fish that were not present in the wild cod [43]. The authors attributed the differences in the proteome to differences during cultivation that may have affected not only the make-up of the muscle in vivo, but also the postmortem muscle conditions (for example, pH) and the abundance and regulation of proteases relevant in postmortem muscle tenderization. A large scale study by Chiozzi and coworkers [44]. comparing the proteome of wild and farmed European seabass with that of wild specimens from the same area in the Mediterranean by label-free multidimensional shotgun proteomics to identify relationships between farming conditions and quality and safety of the fish, confirmed muscle atrophy in farmed fish [44]. The most abundant upregulated proteins in farmed sea bass were some structural proteins and proteins involved in binding and catalytic activities, while the main downregulated proteins also involved catalytic activities and binding.

Optimization of fish diets has been a priority in the aquaculture sector for many years [45]. One ingredient whose incorporation in feed seems to improve growth performance and the humoral immune response of some fish species is β-glucan [46]. Feeding β-glucan to rainbow trout induced an increase in the amounts of tropomyosin isoforms and it lowered those of myosin light and heavy chain isoforms in the proteome of the fillet in treated trout [47]. Evaluation of the effects of partial substitution of fish meal by plant proteins on the fish proteome in different tissues has also been the target of several studies. Thus, partial substitution of fish meal with soybean meal caused an increase in the amount of enzymes involved in protein catabolism and turnover in the liver of rainbow trout [48] and it affected the proteome of gut mucosa in gilthead bream [49].

Reduction of the use of fish meal and oil in aquaculture is a priority [45], which has led to investigate the effects of novel diets on fish physiology where some marine ingredients were substituted by vegetable protein and oils [50]. The inclusion of vegetable oil feed induced a specific response in the intestinal proteome in salmonids, indicating a defense against oxidative cellular stress [51]. Significantly downregulated proteins were those related to oxidative stress and motility, including the myosin light chains, peroxiredoxin-1 and hemopexin-like protein.

Using analytical techniques based on microfluidic electrophoresis and sequencing, some authors have consistently confirmed differences in the protein abundance and/or regulation in fish muscles depending on the production method. Monti et al. [52], using SDS-PAGE, MALDI and ESI MS/MS, and CE for protein identification and relative quantification, showed that the enzymes involved in the metabolism of carbohydrates were upregulated in farmed sea bass muscle (i.e., glyceraldehyde-3-phosphate dehydrogenase and aldolase), while creatine kinase, nuclease diphosphate kinase B and parvalbumin were downregulated, displaying the expected proteome pattern of muscle in farmed fish [52]. Additionally, new protein sources, such as insect meal, have been characterized for aquafeeds by direct comparison through LC-MS/MS analysis [53].

### 3.2. Fish Welfare and Stress Response in Aquaculture

The effects of stress on growth have been studied extensively in animal production and aquaculture [54]. Different chronic stress conditions, such as confinement, overcrowding, repetitive handling, deficient water and diet and hypoxia, affect the welfare and stress response of aquaculture organisms and several proteomic studies have identified robust protein signatures for chronic stress in fish [55]. Elevated cortisol due to long-term stress conditions has a strong impact on the entire organism and is directly linked to the inhibition of muscle growth by inhibiting protein synthesis and increasing protein catabolism to obtain energy from amino acids [56]. The proteome of fish farmed under these stressful conditions displays an increase in the amount of enzymes related to protein catabolism, and a decrease in the amount of the structural proteins, with the latter being degraded to provide energy. Stress-related depletion of the required energy for muscle growth has been shown to lead to muscle atrophy [57].

Farming itself affects the levels of stress the fish suffer, their growth, and the biochemical composition of different tissues and organs, including the liver, brain, and muscle. For instance, exercising in salmonids lowers the levels of aggression and the building up of hierarchies, leading to increased fish welfare and growth [58,59,60]. It is reasonable to assume that these observations would apply to any species with similar behavioral characteristics, i.e., active swimmers with shoaling behavior and schooling responses [61]. Interestingly, while muscle atrophy is provoked by not using the muscle, its use induces both muscle growth and a type of muscle damage due to the need to develop and grow both the activated muscle satellite cells (to regenerate the lesion) and the existing myofibrils that need to increase their volume, i.e., inducing both muscle hyperplasia and hypertrophy [62]. Thus, while normal muscle growth in adult fish will be accomplished by hyperplasia and, mostly, by hypertrophy [63,64,65,66], muscle regeneration and subsequent growth, as observed in exercising fish [62], recapitulates embryonic myogenesis through the activation of satellite cells and the consequent larger contribution from hyperplasia followed by hypertrophy of the muscle fibers [64,67]. This process involves alterations in protein abundance and muscle metabolism to achieve muscle growth [68] and should ultimately lead to an increase in the yield of fillet and to improved welfare of the fish.

One study on sea bream, however, was not able to show consistent differences between the 2-DE patterns of muscle from two natural repopulation lagoons and those of fish from four offshore mariculture plants in Italy by 2-DE, MALDI-MS and LC-ESI-Q-TOF-MS [69]. The similarity between the proteomes of farmed and wild fish would indicate the suitability of the farming conditions and locations. The authors did, however, find significant individual differences in the relative expression of parvalbumin isoforms and of spots corresponding to the myosin-binding protein H (MyBP-H) isoelectric series with no apparent relationship to the length of the fish, its production method or geographical location [69]. Muscle protein patterns obtained by 2-DE analysis showed variations attributed to different factors; for example, acclimation to higher water temperature significantly increased the amount of the warm temperature acclimation-related protein-65 isoforms, and the ratio of structural proteins vs. glycolytic enzymes increased as fish grew larger [69]. This work is particularly interesting because it shows that it is possible to achieve offshore-farmed gilthead sea breams of commercial size whose protein expression profile is comparable to that of wild fish [69].

Discovery proteomics has been applied to improve our understanding of the mechanisms implicated in skeletal deformities [70]. Analysis of how preslaughter stress affects the postmortem processes in gilthead seabream muscle was performed by 2-DE and MALDI-TOF-TOF MS [70]. Moreover, 2-DE followed by LC-MS/MS of grass carp gills uncovered alterations in the metabolic pathways after hypoxic stress [71], some of which were involved in energy generation, metabolic, immunity and oxidative processes and proteolytic activities. It must be emphasized that the improvement of farming practices, leading to minimizing chronic stress and preserving fish welfare not only is one of the primary challenges for fish farmers, it is also a demand by European consumers.

The Pacific geoduck clam is one of the species whose farming is seeing a successful bloom and, consequently, a species under study to improve its production [72]. Proteomic studies on how geoduck production may be impacted by conditions susceptible of being modified by ocean acidification, such as pH and temperature were performed by Spencer et al. [73]. The results showed that the amounts of heat shock protein 90-α, puromycin-sensitive aminopeptidase and tri-functional-enzyme β-subunit as well as shell growth, kept a negative correlation with the average temperature and a positive one with the amount of dissolved oxygen. That indicates that geoducks may be more resistant to acidification under natural conditions and more susceptible to variations in the concentration of dissolved oxygen and the temperature of the water.

### 3.3. Food Safety in Aquaculture

Aquaculture has the capacity to provide food for millions of people over the world, however inappropriate facility management may severely damage aquatic ecosystems and affect health risks to consumers through contamination with environmental or human-made hazards. Moreover, several pathogenic microorganisms can be found in the aquatic environment with the potential to negatively affect, not only aquatic life, but also human health. In the last decade, the risk of dissemination of infectious or toxic agents and the occurrence of disease outbreaks has risen mainly due to increases in the following factors: (i) intake of raw or scarcely processed seafood; (ii) international trade of aquaculture products; (iii) suboptimal monitoring methodologies; (iv) alterations in ecological stability; and (v) contamination and climatic change [74]. Food safety in the aquaculture sector is of crucial relevance to avoid health hazards that can be biotic (bacteria, allergies, parasites, virus or harmful algae blooms) and abiotic (aromatic hydrocarbons, dioxins, heavy metals, plastics) [75]. To control and minimize the presence of hazards, the FAO implemented a code of practice for aquaculture products [1], where the handling of fish is presented according the requirements of HACCP.

To make the reading of this review more comprehensible, the authors have decided to divide the hazards to which consumers of aquaculture products are exposed into two general groups: biotic hazards and abiotic hazards.

#### 3.3.1. Food Safety in Aquaculture: Biotic Hazards

Foodborne poisonings are a relevant cause of mortality and morbidity which result from drinking water or eating food contaminated with such pathogens as viruses, bacteria and parasites, and their toxins.

Regarding biotic hazards, there are two main groups of bacteria that affect food products of aquaculture: those naturally present in its habitat (*Aeromonas* spp., *Clostribuim botulinum*, *Listeria monocytogenes*, *Vibrio cholerae,* and *Vibrio parahaemolyticus*) and those derived from environmental contamination (Enterobacteriaceae, *Escherichia coli,* and *Salmonella* spp.) [76]. Additionally, *Staphylococcus aureus* can infect aquaculture species during management due to inadequate hygiene conducts of operators in the processing factories [77,78].

Proteomics has been applied to the detection and identification of bacterial species from aquaculture products both after their direct detection in fish products, or after their isolation and growing in different culture media. For instance, MALDI-TOF-MS techniques enabled us to achieve mass spectral fingerprints of *Vibrio* spp., a Gram-negative bacteria causative of gastrointestinal diseases in humans after ingestion of poorly cooked infected seafood, such as seabream and mollusks [79,80]. The exhaustive proteome and transcriptome data analysis obtained by the study of Li and coworkers by iTRAQ coupled to MRM provided some critical protein signatures for the study of the regulatory mechanisms of the intestinal mucosal immunity in grass carp (*Ctenopharyngodon idella*) against *Vibrio mimicus* [81]. Vaccination altered the regulation of 5339 genes and of 1173 proteins in the grass carp intestines. The conclusions of the study suggest that the integration of the five activated immune-related pathways is relevant to the improved immune response of the intestinal mucosal in immunized carp. MALDI-TOF MS analyses have been performed to obtain available reference spectral libraries for diverse bacterial strains isolated from seafoods [82]. Recently, the open MALDI Biotyper library (Bruker MALDI Biotyper) allowed for the precise identification of 75 pathogenic bacterial isolates [83].

Targeted proteomics applications in the field of pathogenic bacteria have increased substantially in recent years. For example, both SRM and PRM have produced sensitive quantitative results about the proteins associated with bacterial infection, particularly in the fields of clinical diagnosis and antibiotic resistance [84]. Thus, iTRAQ-based quantitative proteomics followed by MRM studies were used to contrast the differentially regulated proteins of *Aeromonas veronii*. This bacterium, a Gram-negative virulent pathogen associated with infections in freshwater fish species and mammals, is capable of adhering to biotic and abiotic surfaces surrounded by the extracellular matrix produced by the resident microorganisms. The study, which used an in vitro biofilm model [85], showed that the upregulated TonB protein increased the nutrient absorption capacity, and the enolase gene was involved in the regulation of multiple pathways, leading to enhancement of the bacteria’s ability to undergo invasion and metastasis. These changes may be the principal cause for the capability of *A. veronii* to create biofilms and its increased dissemination.

Protozoan parasites, such as *Ichthyophthirius multifiliis*, cause important economic losses to the aquaculture sector. Upon exposure to the parasite, LC-ESI-MS/MS revealed the differential regulation of some immune-related signal transduction proteins in the skin mucus of common carp [86]. Multiple lectins and several serpins with protease inhibitor activity were likely implicated in lectin pathway activation and regulation of proteolysis, indicating that these proteins support the carp innate immune system and the preventive characteristics of the skin mucus.

Virus infections can decimate the production in fish and shrimp farms. Among the latter, white spot syndrome virus is currently one of the most serious global hazards. A protein interactomics map for the white spot syndrome virus has been produced by means of a co-immunoprecipitation assay (co-IP) from a yeast two-hybrid approach [87].

Harmful algal blooms (HABs) generate shellfish poisoning toxins that affect aquaculture, particularly mussel farming. Gel-based proteomic approaches were used to distinguish and identify nontoxic dinoflagellates from the toxic dinoflagellate *Alexandrium tamarense* [88]. An alternative approach consists of the generation and application of monoclonal antibodies directed against intracellular antigens of the toxic dinoflagellate *Alexandrium minutum* as described by Carrera et al. (2010) [89]. Recent proteomic studies have contributed to enlarge the volume of data in sequence databases suitable to identify how the proteomes of HABs are modulated by physiological parameters and in response to changes in the environment, such as climate change [90]. In that regard, Piñeiro et al. (2010) [91] reviewed the application of proteomics methods to study the effects of climate change on the quality and safety of wild and cultivated seafood products.

Proteomic studies and systems biology analysis of allergenic proteins have also been critical determinants for the evaluation of the quality and safety of wild and cultivated fish and crustacean food products [92]. The major allergen identified in fish is β-parvalbumin. A rapid strategy for the detection of fish β-parvalbumin in fish products was performed by targeted proteomics using SMIM [93]. On the other hand, tropomyosin is the major allergen in shrimp and mollusks. Proteomic profiling of the allergen tropomyosin was performed to obtain the full amino acid sequence in a Q-TOF instrument [94]. Recently, the impact of EDTA-enriched diets on farmed fish allergenicity was studied by 2-DE [95].

#### 3.3.2. Food Safety in Aquaculture: Abiotic Hazards

Abiotic hazards in aquaculture have been extensively studied in mollusks exposed to contaminants in polluted areas. Discovery proteomics studies have been mainly performed for environmental assessment and marine pollution monitoring using the digestive glands of mussels *Mytilus galloprovincialis* by 2-DE and MS [96]. Bottom-up proteomics approaches on mussels exposed to fresh fuel and weathered fuel in a laboratory experiment that attempted to mimic the effects of the Prestige’s oil spill were performed by 2-DE and MS [97]. Moreover, 2-DE and MALDI-TOF/TOF MS analyses have also been applied to the identification of differentially regulated proteins in the gonads of the oyster *Crassostrea angulata* after HgCl_2_ contamination [98]. The first shotgun proteomics analysis of mussels after exposure to pharmaceutical environmental contaminants, such as propanolol, was performed by Campos et al. (2016) [99].

To assess complex field contamination, targeted proteomics using SRM methodologies was applied to the quantification of dozens of protein biomarkers in caged amphipods (*Gammarus fossarum*) after in situ exposure to several aquatic environments [100]. The work detected some of the previously identified and currently well-established protein biomarkers for amphipod crustaceans, such as the detoxification/antioxidant enzymes glutathione S-transferase, acetylcholinesterase, catalase, superoxide dismutase and some digestive enzymes [100].

Nanoparticle pollution is a recent issue of concern that has also been addressed by proteomic techniques. Thus, targeted proteomics has shown that the ionic form of Ag impacted on the growth of *Pseudomonas* spp. more strongly than did the nanoparticulate form of Ag in a bacterium isolated from waters in a region where fish is farmed for human consumption [101]. Possessing broad-spectrum antimicrobial properties, silver nanoparticles (AgNPs) are widely used in textiles and medical drugs. Approximately 20–130 tons of ionic silver (Ag^+^) have been predicted to reach EU freshwaters annually, mostly due to leaching of ionic AgNPs from biocidal plastics and textiles. Proteomic analysis using SWATH-MS allowed the identification of 166 proteins affected by exposure to the nanoparticulate form of Ag, which also impacted on the growth of *Pseudomonas* spp. The form of Ag induced different adaptive responses in the metabolic, stress, and energetic pathways in *Pseudomonas* spp., and proteins affected were transmembrane transporters, chaperones, and proteins related to the metabolism of carbohydrates and proteins, indicating their potential value as biomarkers of the stress induced by Ag^+^ and/or AgNPs. Among all the modified proteins, 59 had their content significantly changed by one or both forms of silver. In view of the evidences obtained in these studies, we believe that nanoparticle pollution should be considered an emergent hazard in waters with aquaculture production.

### 3.4. Antibiotic Resistance in Aquaculture

Antibiotics are natural and synthetic compounds that kill bacteria and have been heavily used and abused in aquaculture for over 50 years [102] not only to treat and prevent infections, but also to promote growth. Fortunately, success in the development of vaccines for the most relevant infections and in the implementation of vaccination programs has greatly reduced their use in some countries (e.g., Norway) although it is still a very serious problem in other countries and in the breeding of some species.

The abuse in antibiotic treatments has provoked the development and spreading of bacterial resistance and the appearance and expansion of multidrug-resistant strains in such a way that the aquatic environment has become an important reservoir of antibiotic resistance genes/proteins (ARG/Ps) and a route for their dissemination and potential transmission to human pathogens. Until now, five main mechanisms of antibiotic resistance have been accurately recorded due to the related development of resistance to drugs that (a) deteriorate enzymes, (b) bypass target pathways, (c) change antibiotic focus sites, (d) alter the penetrability of porins, and/or (e) trigger flow systems [103].

Proteomics techniques have the potential to significantly contribute to increase the knowledge about molecular mechanisms related to antibiotic resistance [104]. In the last five years, metagenomics and metaproteomics have been applied to identify correlations between the “resistome” (the antibiotic immunity genes/proteins) and the transmission of ARG/Ps from natural microflora to human pathogenic microorganisms, which could become a serious health issue. Conventional methodologies to evaluate water quality had been used to analyze marine sediments close to aquaculture farms, evidencing that the native “resistome” had been enriched by the use of antibiotics at the farming sites, although the findings were restricted to only a group of genes/proteins [5].

Proteomic studies on antibiotic resistance of fish- and shellfish-borne bacteria have largely been performed on *Aeromonas* spp., because this pathogen is responsible of hemorrhagic septicemia and hemolytic diseases in aquaculture, which cause large financial losses to farmers. Some of these studies are described below.

Tetracyclines are commonly used antibiotics comprising the monocyclines group, doxycycline, and chlortetracycline (CTC), and they are very efficient against both gram-positive and gram-negative microbes. In aquaculture, tetracycline resistant *Aeromonas hydrophila* (a notorious pathogen causing infections in many relevant wild and farmed species including carp, shellfish, grass carp and shrimp) has been confirmed by different proteomics analyses. Comparison between the fitness and acquired resistance to CTC in an *Aeromonas hydrophila* biofilm by TMT-labeling-based quantitative proteomics indicated an increase in translation-related ribosomal proteins in both cases and an increase in proteins involved in fatty acid biosynthesis only in biofilm fitness, while proteins involved in other pathways were less abundant in acquired resistance biofilm. Targeting the up-regulation of fatty acid biosynthesis, the authors found that a mixture of CTC and triclosan (a fatty-acid-biosynthesis inhibitor) had a more powerful antimicrobial effect than either one of them alone. This information is highly relevant in the fight against this pathogen when forming biofilms, which are always a challenge in seafood farming and processing [105].

Two different quantitative proteomic studies, using dimethyl labeling and label-free methods, performed on the same year as the previous work, were conducted to examine the differential regulation of proteins in response to several doses of oxytetracycline (OXY) in *A. hydrophila* [103]. The results showed an increase in translation-related proteins, although the amount of many central metabolic-related proteins decreased upon OXY treatment and, also, antibiotic sensitivity seemed to be significantly inhibited by numerous external metabolites when they were compounded with OXY antibiotics.

In 2018, Li et al. published a quantitative proteomics experiment based on iTRAQ methodology, to compare proteins differentially regulated in CTC-resistant *A. hydrophila* and in control strains [106]. The majority of the detected differentially regulated proteins were involved in key energy biosynthesis pathways, such as metabolic and catabolic processes, transportation and signal transduction. Chemotaxis-related proteins were downregulated in CTC-resistant strains, but exogenous metabolite addition increased bacterial susceptibility in *A. hydrophila*. In addition, Elbehiry and colleagues described the application of MALDI-TOF MS for the discrimination of the *Aeromonas* genus from meat and water samples, with a spotlight on the antimicrobial resistance of *A. hydrophila* [107].

Recently, another proteomic study using 2-DE and MALDI-TOF/TOF analysis was conducted by Zhu and coworkers with multidrug resistant (MDR) and sensitive *A. hydrophila* strains to find differences in the regulation of proteins [108]. The work showed that, in the sensitive strains, proteins engaged in glycolysis/gluconeogenesis and antibiotic biosynthesis were up regulated in the MDR strain, while those involved in biosynthesis of secondary metabolites, cationic antimicrobial peptide resistance, metabolic processes related to carbon regulation and bacterial metabolism were downregulated. Other proteomics approaches have been used to obtain knowledge about antibiotic resistance in other pathogenic genera, such as *Edwardsiella*, a gram-negative microorganism that also generates hemorrhagic septicemia in a broad number of cultivated fish species, including yellowtail carp and eels. As in the case of *Aeromonas*, the antibiotic resistance status of *Edwardsiella tarda* is of high relevance for seafood safety, particularly when the bacterium is forming biofilms. Sun and coworkers, used iTRAQ-based quantitative proteomics and high-resolution LC-MS/MS to analyze the differential protein regulation of *E. tarda* in response to OXY stress in biofilms [109]. Their work showed a total of 281 modified proteins, 193 of which were downregulated and 88 upregulated. As *A. hydrophila* in biofilms, many ribosomal proteins were upregulated in response to the stress in *E. tarda*, while treatment with OXY increased the amount of Uvr C, a member of the UvrABC system that plays an important role in multiple antibiotic resistance processes.

iTRAQ and LC-MS/MS were used in conjunction to evidence the differential proteome of the ampicillin-resistant LTB4 (LTB4-RAMP) strain of the gram-negative facultative aerobic bacteria *Edwardsiella piscicida* and showed that a depressed P cycle seemed to be a characteristic of the differential proteome in the LTB4-RAMP [110] strain, leading the authors to conclude that the depressed P cycle caused the ampicillin resistance in *E. piscicida*.

The above research works in aquatic organisms, using iTRAQ technologies, seem to indicate that the acquisition of antibiotic resistance involves chemotaxis, energy metabolism, biofilm characteristics and external membrane proteins, as well as networks of proteins associated to antibiotic resistance.

Finally, “reprogramming proteomics” needs to be developed in a general manner to revert an antibiotic-resistance proteome to an antibiotic-sensitive proteome for the control of antibiotic-resistant pathogens [104].

## 4. Concluding Remarks and Future Directions

As presented in this review, proteomic approaches help to characterize some of the principal issues associated to farming conditions and to address some of the main challenges in aquaculture, such as dietary management, fish welfare, stress responses, food safety and antibiotic resistance.

Proteomics helps to elucidate how dietary management in the aquaculture sector influences the production, growth, immunity and wellness/welfare of living aquaculture organisms and assists in the selection of optimal diets. A large number of publications have shown that the composition of the feed influences the fish muscle nutritional value and its proteome. Efforts have been made to discover protein markers for such quality traits. In addition, various proteomics investigations have been published on the identification of robust protein signatures for fish chronic stress. From this perspective, amelioration of aquaculture conditions to reduce chronic stress during farming and maintain fish welfare is one of the principal issues that can be addressed with discovery proteomics. Additionally, innovative fast targeted proteomics workflows have demonstrated the rapid detection of fish allergens, parasites and microorganisms in aquaculture. The characterization of species-specific peptides by MS/MS-based proteomics and their monitoring by targeted proteomics demonstrated the adequacy of these approaches for food safety control, enabling the differential detection of several hazards in the aquaculture sector. In this way, the utilization of rapid sample preparation methods, combined with sensitive and accurate MS for both the discovery and targeting of fish quality and safety biomarkers, may enhance quality control and safety in aquaculture. Moreover, proteomics offers a more holistic point of view on the molecular mechanisms of antibiotic resistance in the aquaculture sector and it can be directly linked to the metagenomic/metaproteomic approaches that are being applied to the study of a new concept known as the resistome, a current challenge of high relevance that needs an effective and rapid response and that may be elucidated through proteomics techniques.

As the proteins are considered the principal functional macromolecules in all biological systems, we consider that proteomics strategies and their associated techniques can offer several advantages compared with other methodologies for the study of the impact of farming conditions on food quality and safety in aquaculture products. This is the case primarily because, with those methodologies, it is possible to identify and directly quantify protein/peptide signatures without the necessity of inferring conclusions based on other approaches such as genomics tools. Secondly, the benefits of proteomic analysis may be adapted for fish products with short shelf-life. Finally, the current advances in proteomic methodologies allow for the implementation of precise methods that may be useful for routine control test with a potentially lower cost and in a relatively short estimated time (<30 min).

Lastly, the development and practical implementation of new advances based on protein arrays, microfluidics, and biosensors to the aquaculture sector offers a promising research area in which the results of proteomic studies can be established for the routine control test and diagnosis of fish products. We also assume that the digitalization of these new devices may be relevant to the aquaculture industry and control authorities in the next several years and may supply rapid monitoring information to effectively drive decision enforcing by the industry and authorities.
